# Covid-19: current knowledge, disease potential, prevention and clinical advances

**DOI:** 10.3906/biy-2005-29

**Published:** 2020-06-21

**Authors:** Aftab ALAM, Mohd Faizan SIDDIQUI, Nikhat IMAM, Rafat ALI, Md. Mushtaque, Romana ISHRAT

**Affiliations:** 1 Center for Interdisciplinary Research in Basic Sciences, JMI University, New Delhi India; 2 International Medical Faculty, Osh State University, Osh City, Kyrgyz Republic Kyrgyzstan; 3 Institute of Computer Science & Information Technology, Department of Mathematics, Magadh University, Bodh Gaya, Bihar India; 4 Department of Chemistry, School of Physical and Molecular Sciences, Al-Falah University, Dhauj, Faridabad, Haryana India

**Keywords:** COVID-19, transmission rate (Ro), mortality rate, vaccines, antivirals, financial crisis

## Abstract

The top priority of any nation is to lead the nation towards prosperity, progress, and economic growth, confronting several challenges and concerns arisen from global situations. The sudden outbreak of any disease defies the health care systems and economy of nations. COVID-19 is one of the viral diseases which broke out in Wuhan city of China in 2019. COVID-19 outbreak intermittently prevailed all over the world. It exposes the fragility of the established health care systems across the world in spite of comprising modern science and technology. Unfortunately, there is no chemotherapeutic agent in the regimen of antiviral drugs or no vaccine available to curb this infectious disease. As a consequence, this deadly infection has prevailed all over the world. The antiviral drugs used for viral diseases excluding COVID-19 infection are Ramdesvir, Favipiravir, and Ribavarin, and antimalarial agents (Chloroquine & Hydroxychloroquine) are being administered to the patients for redemption of this infection. Fortunately, these existing drugs have been found clinically active and are being used. In this review, we present the current scenario and status of epidemiology, diagnosis, treatment, vaccine development for COVID-19, and its impact on the socio-economic structure.

## 1. Introduction

Coronavirus is one of the lethal viruses that human race have ever seen. Indeed, coronavirus is a single-strand RNA virus that utilizes host cells for the replication of their genome (Bhupender et al, 2020; Ju et al., 2020) the immediate emergency therapy is not available for the treatment of this disease, leading to widespread fear of infection and has created social issues for infected peoples. The SARS-CoV-2 genomes encode a protein which is known as RNA-dependent RNA polymerase (RdRp). Through this, new copies of RNA virus are transcribed in the host cells by viral genome. Thus, replication of RNA inhibition is quite important to control infections. These viruses have been categorized into four types as alpha-coronavirus, beta-coronavirus, delta-coronavirus, and gamma-coronavirus. Among these, α and β CoVs are known to be the causative agents of infections in humans (de Wilde et al., 2017a). HCov 229E, NL63, OC43, and HKU1 are the four globally endemic human coronaviruses (*HCoVs*) which are accountable for 10% to 30% of upper respiratory tract infection in adults. A large variety of ecologically diverse coronaviruses are mainly seen in bats, which are also the reservoir for many of these viruses (de Wit et al., 2016). Coronaviruses have been named for the crown-like spikes on their surface which are glycoprotein, critical for binding of host cell receptors and are believed to represent a key determinant of host range restriction. The *HCoVs* earlier received quite low attention due to their mild effect in humans. However, in 2002, it emerged as an important human pathogen when the cases of severe atypical pneumonia emerged in China, causing a large-scale epidemic. More than 774 deaths in total, and more than 8000 infections caused worldwide concern. This new disease was further named as severe acute respiratory syndrome CoV (SARS-CoV), and a beta-HCoV, named SARS-CoV, was identified as the causative agent. Over a decade in 2012, Middle East respiratory syndrome (MERS-CoV) caused panic and a persistent epidemic in the middle eastern countries (Cheng et al., 2007; Chan et al., 2015). In both cases, there was history of human–animal contacts, so zoonotic transmission of HCoVs was highly suspected, and a consensus emerged that bats were the natural hosts and the virus transmitted into another amplification mammalian host [masked palm civet (*Pagumalarvata*) for SARS-CoV and dromedary (*Camelus dromedarius*) for (MERS-CoV)] before crossing species barrier to infect humans. 

Coronavirus disease 2019 is a contagious disease caused by SARS family virus SARS-CoV-2. The initial symptoms of COVID-19 are coughing, throat infection, fever, breath shortness, muscle pain, and fatigue, and later the symptoms become severe pneumonia, kidney failure, and acute respiratory distress syndrome (ARDS). The incubation period for exposure to onset is 2 to 14 days according to WHO and the US Center for Disease Control and Prevention. A full genomic sequence of COVID-19, known as SARS-CoV-2, was released by the Shanghai Public Health Clinic Center (SPHCC) and School of Public Health (SPH) and their collaborators to public data in this havoc outbreak response. We can access the complete genome sequence of SARS-CoV-2 (Wuhan-Hu-1) from GenBank (Accession no: NC_045512/MN908947.3).

## 2. Demography: global health emergency and current status

On December 1, 2019; numerous pneumonia cases were reported to the World Health Organization (WHO) by the Chinese Health Authority in Wuhan city, Hubei Province, China (WHO, 2020a). The earliest signs of these patients include fever, malaise, dry cough, and dyspnea. The CT scan of these patients shows pneumonia with abnormal findings and COVID-19 was detected in virological tests of these patients. In just 4 months, the COVID-19 that originally emerged in the Wuhan city of China poses a major public health issue and governance challenges. The disease has now spread throughout the world. There was an unknowingly large spread of the infection through human-to-human transmission among the national and international travelers during the Chinese New Year holidays, which resulted in the mobility of the virus globally. 

**Figure 1:**
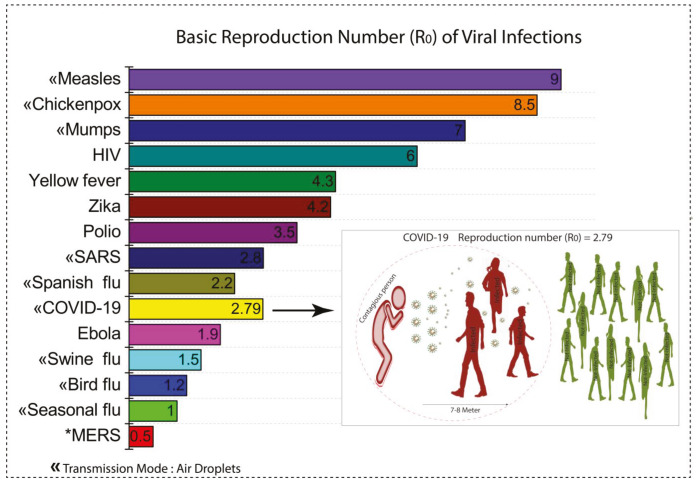
Average basic reproduction number (R0) of most common viruses.

COVID-19 is affecting more than 210 countries and territories, and some countries are more affected, such as the USA, Spain, Italy, France, Germany, UK, Turkey, Iran, and China. The estimated mortality rate is 3.4% by WHO as of March 3. The basic reproduction number (R0) for COVID-19 so far seems to between 2.5 and 2.79 although the current R_0_ value might be biased because of insufficient data. The current R0 value of COVID-19 is much lower in comparison to other viruses like chickenpox, mums, and SARS (Figure 1) but many experts have suggested that COVID-19 may be far more infectious than the flu. If we go through the stats, we will notice a significant change in the mortality rate of most of the countries over time. Also, the accurate data of the mortality rate of different countries are still unavailable and now the logarithmic curve of per day new cases and deaths from the previous active cases could be considered one of the reasons for the escalation in the mortality rate of those countries. However, if we look at the macroimage of the complete stats, it will show a consistent trend of increase in the number of per day new cases marked by many countries like the USA, Spain, Italy, UK, Russia, France, Germany, Turkey, Brazil, and Iran. However, it is too early now to consider these data to compare the current mortality rates and demographics of countries, since each of these or other countries are in the different stages of the COVID-19 pandemic. For instance, it is unscrupulous to compare the current mortality rate of Spain, USA, Italy, and France with that of India, Pakistan, and Bangladesh, as the countries in these categories are currently having different stages of the outbreak. In addition, many other factors affect the stats of mortality rate are age, smoking, and comorbidities in many countries [6]. As of May 8, 2020; more than 3.9 million infected cases with 270 thousand deaths around the globe have been reported. At present, no vaccine or specific treatment is available, which makes it more deadly.

## 3. The coronavirus disease (COVID-2019): SARS-CoV-2 virus

The outbreak of the respiratory syndrome causing a major epidemic by the coronavirus family was named temporarily as 2019 novel coronavirus (COVID-19)^1^1Coronavirus disease (COVID-19) outbreak. [online] Website: https://www.who.int/westernpacific/emergencies/covid-19 [accessed 7 May 2020].. Here the word novel refers to emerging human disease by this coronavirus family which required further studies (Chen et al., 2020). Further, it was revealed in the genetic analysis of the COVID-19 that the virus was noticeably similar (with up to 88%) to the bat-derived (SARS). Regardless the variation of the amino acid at a few key residues, homology modeling showed that COVID-19 has a similar receptor binding domain structure to that of SARS-CoV (Lu et al., 2020). Similarly, the catalytic sites of the four COVID-19 enzymes that could represent antiviral targets are remarkably conserved and have mutual sequence similarity with the corresponding enzymes of *SARS* and *MERS* (Li and De Clercq, 2020; Liu et al., 2020). The International Committee on Taxonomy of Viruses (ICTV), which is responsible for officially classifying and naming of the family Coronaviridae, is the Coronavirus Study Group (CSG). This novel virus was formally recognized by CSG as a sister to the (SARS-CoVs) and assigned it as (SARS-CoV-2) (Gorbalenya et al., 2020). Further, the WHO and ICTV confirmed and announced the name of the virus as (SARS-CoV-2), and its disease outbreak as COVID-19, which refer to the disease or illness caused by this SARS-CoV-2. It is a functional naming convention with a standard format and can be used for any future outbreak of coronavirus^2^2Cohen J. COVID-19: Why names of new infectious diseases matter. [online] Website: https://www.forbes.com/sites/joshua-cohen/2020/02/13/covid-19-why-names-of-new-infectious-dis-eases-matter/#1f1a7b16ac63 [accessed 7 May 2020]..

## 4. Clinical manifestation and complication 

The most common symptoms of COVID-19 at the onset of illness were fatal pneumonia, fever, headache, cough, sputum production dyspnea, myalgia, fatigue. Older patients (aged >60 years) with comorbidities appear to be more vulnerable to becoming severely ill with the infection and had more systemic symptoms. The incubation period for the infection with SARS-CoV-2 ranges from 2 to 14 days after exposure. The zoonotic coronaviruses were identified as severe human pathogens in the previous outbreak of (SARS) at the beginning of 2003 and Middle East respiratory syndrome in 2012 (Chan et al., 2015). However, the clinical presentation, symptoms, and complication in both these outbreaks share many resemblances with the recent Coronavirus (COVID-19). The infection has been associated with complications like organ dysfunction (acute respiratory distress syndrome [ARDS], acute cardiac injury, acute kidney injury, septic shock) and severe cases result in the death of the patient (Wang et al., 2020). Besides, many countries, including China and India, have reported the asymptomatic cases of COVID-19. The asymptomatic patients cause concerns of a next wave of infections amid the relaxation of stringent measures in the country initiated to contain the lethal disease.

## 5. SARS-CoV-2 transmission in humans 

The majority of the initial infected cases were linked to Huanan seafood and wild animal wholesale market, which has been marked as the epidemic center by the Chinese health authority.  It is believed that the virus possibly originated from horseshoe bats in China and then was transmitted to other animals which are usually eaten by humans. Also, based on their research, South China Agricultural University claimed that the pangolin too acts as the key source of novel coronavirus (COVID-19) after the genetic comparison of COVID-2019 taken from the infected humans and animals in their research and they noted the similarity of 99% in their genetic sequences (Cyranoski, 2020). The following common modes of infection transmission have been reported:

### 5.1. Through animals

The majority of COVID-19 infection cases shared the history of zoonotic transmissions like *SARS-CoV* and MERS-CoV because many of the first individuals found to be infected by the virus were workers at the Huanan seafood market. According to the US-based Center for Disease Control and Prevention (CDC), the Wuhan coronavirus likely started from a “spillover” that occurs when the virus was passed from animal to human. These spillovers are reported to ensue through close human-to-animal contact, particularly in wet markets (Li et al., 2019). In the same way, to justify the transmission through animals, a “snake theory” was very popular. A study claims there is evidence that COVID-19 came from snakes sold at the Wuhan market. They found that the virus appears to be a mix or “recombination” of two coronaviruses, Homologous recombination within the spike glycoprotein of the recently recognized coronavirus may increase cross‐species transmission from snake to human (Ji et al., 2020). It has shown that approximately CoVs have been spotted in bats in China. A large amount of evidence suggests that there is a link between COVID-19 and other known CoVs in bats, specifically in Rhinolophus bat subspecies which is abundant and mostly present in Southern China, and across Asia, the Middle East, Africa, and Europe regions (WHO, 2020b; Wang et al., 2018). However, few investigators have advocated that the Huanan seafood market may not be the original source of the viral transmission to humans (Cohen, 2020).

### 5.2. Human-to-human transmission

Potential asymptomatic transmission and human-to-human transmission has been reported for most of the infection within the family members, office crew, healthcare practitioners, through air travels, and from a person who had recently travelled or repatriated from infection-prone countries (Chan et al., 2020). It is a vertical transmission when an infectious agent is transmitted from mother to baby during the period immediately before and after birth. The first known case of vertical transmission in China was reported on 5 Feb 2020. However, it is still unclear whether the newborn was infected with SARS-CoV-2 through vertical transmission or contracted the virus from the hospital environment. Still, much research and information are needed in this case.

### 5.3. Fecal transmission

Recently, Chinese researchers found that COVID_19 was present in the feces of infected people, which may help to elucidate why it spread so fast on cruise ships (Diamond Princess). Also with a negative result in respiratory tract sample and presence of SARS CoV-2 RNA in patients’ fecal sample at an average of 11 days might be confirmed through the stool which can further contaminate the hands, food, and water bodies^3^3Gale J. Fecal transmission may be behind coronavirus’s rapid spread. [online] Website: https://www.bloomberg.com/news/articles/2020-02-20/fecal-transmission-may-be-behind-corona-virus-s-rapid-spread [accessed 7 May 2020]..

## 6. Transmission stages

In stage 1, the cases are imported from infection-prone countries and the virus does not spread locally. At this stage, it is easy to test, track, and treat the patients. When it comes to stage 2, the virus could be transmitted through contact with affected people. Here, the most affected people are their family members, friends, and people who come in contact with them. The third stage is the community transmission where an individual becomes infected without any travel history to affected countries, and nobody knows the exact source of the virus. This stage is extremely infectious and difficult to control in densely populated countries like India, Bangladesh, and China. Stage 4 is the worst form of disease transmission which is totally out of control, such as it was in the US, Spain, Italy, France, and Germany.

## 7. Vaccines and antivirals: current status and future directions

At present, no vaccine or specific treatment is available; however, Chinese health authorities endorsed the use of u1d14 (TCM) for infected individuals with COVID-19. Additionally, many drug companies are claiming to have developed like the India-based pharmaceutical firm Zydus Cadila^4^4Zydus Cadila looks to expedite Covid-19 vaccine development. [online] Website: https://www.pharmaceutical-technology.com/news/zydus-cadila-covid-19-vaccine/ [accessed 7 May 2020] which has recently started DNA vaccine development program against the COVID-19 (Pharmaceutical Tech., 2020). Moreover, the Health and Human Services (HHS), USA, Sanofi Pasteur and Johnson & Johnson have taken the initiative to develop vaccine and therapeutics to use against COVID-19^5^5Soucheray S. HHS partners with drug makers on COVID-19 vaccine, drugs. [online] Website: https://www.cidrap.umn.edu/news-perspective/2020/02/hhs-partners-drug-makers-covid-19-vaccine-drugs [accessed 7 May 2020]
66SARS treatments could help with the new coronavirus. Why were they shelved? [online] Website: https://fortune.com/2020/02/18/coronavirus-sars-vaccine-development/ [accessed 7 May 2020]. In a similar way, the Taiwan-based company “Medigen Vaccine Biologics Corp (MVC)” recently publicized that it has teamed up with the US National Institutes of Health (NIH) to develop a vaccine against COVID-19^7^7Taiwan firm teams up with US to develop Covid-19 vaccine, 2020. [online] Website: https://www.thestar.com.my/news/re-gional/2020/02/17/taiwan-firm-teams-up-with-us-to-develop-covid-19-vaccine [accessed 7 May 2020]. On 21 Feb 2020, a researcher from Tulane University (US) also announced to have developed a vaccine, and named it COVID-19^8^8Tulane University to develop vaccine for novel coronavirus. [online] Website: https://www.pharmaceutical-technology.com/news/tulane-univeristy-coronavirus-vaccine/ [accessed 7 May 2020].

In the era of advanced and effective vaccines and antiviral therapeutics, COVID-19 again proved that we are nothing in front of the nature and it presents a major threat to human beings. However, many research projects are being run to develop better treatments.

### 7.1. DNA vaccination

The genetically modified DNA is injected into the cells so it can directly manufacture an antigen which gives a defensive immunological response.

### 7.2. Live-attenuated investigational vaccine (LAIV)

The LAIV can protect from COVID-19. In this type of vaccine, the virus has been incapacitated so that it cannot cause disease.

### 7.3. Investigational mRNA vaccine

The mRNA has been introduced as a very flexible and efficacious stage to deliver vaccine antigens and therapeutic proteins and can be easily altered to express most viral proteins and can be produced efficiently (Alam et al., 2017).

### 7.4. Plasma protein therapies

Patients who recovered from COVID-19 still have antibodies in their bodies. By injecting these antibodies into a new COVID-19 patient (immunity transfer) can help to overcome it^9^9Saplakoglu Y. Blood from cured coronavirus patients could help treat infection. [online] Website: https://www.livescience.com/blood-plasma-cured-patients.html [accessed 7 May 2020]^10^10Novel Coronavirus(2019-nCoV) (Situation Report – 22 ). [online] Website: https://apps.who.int/iris/handle/10665/330991 [accessed 7 May 2020]
.

### 7.5. The spike glycoprotein

It is the major surface protein that the virus uses to bind to a receptor and it acts as a doorway into the human cell. The viral membrane fuses with human cell membrane allowing the genome of the virus to enter into the human cell and begin infection; if we can prevent attachment and fusion, we can prevent the entry of the viruses (Cohen, 2016). Thus, these spike proteins can be new targets in the fight against COVID-19.

### 7.6. COVID-19 Neutralization

We can control COVID-19 by recognizing its transmission mechanism in humans. The elucidation of the molecular crosstalk that links COVID-19 interaction with the host cell receptors that cause infections, replication of virus and involved signaling pathways in the immune response will give the basic understanding about its pathogenesis.

### 7.7. Chemotherapeutic agents

**Figure 2:**
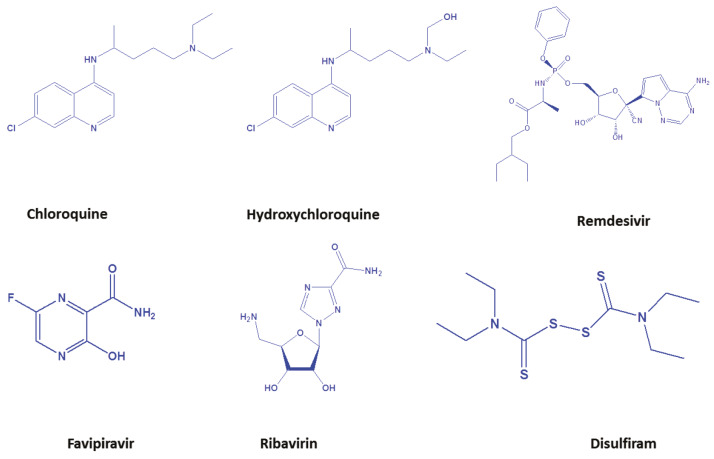
The chemotherapeutic agents exploited to curb the disease produced by COVID-19.

Although there are no chemotherapeutic agents available to curb this menace, few agents are being exploited. Some of them are antiprotozoal regimen and others are antivirals. Chloroquine, Hydroxychloroquine, Sofosbuvir, Remdesivir, Favipiravir, Disulfiram, Ritonavir, Lopinavir are chemotherapeutic agents (Figure 2) that are being exploited to circumvent novel-Covid-19. Until the discovery of drugs for the virus, these existing drugs are Hobson’s choice for the treatment of the corona virus. It is well known that corona viruses are RNA viruses which utilize the host cells for the replication of their genome. The corona virus genomes encode a protein which is known as RNA-dependent RNA polymerase (RdRp). Through this, new RNA copies are transcribed into the host cells by viral genome. Thus, replication of RNA inhibition is quite important to control infections. In this pursuit, RNA-dependent RNA polymerase (RdRp) is one of the most significant targets to discover new chemotherapeutic agents. Nucleosides and nucleotides of RNA-bases are being exploited as pro-drug to inhibit RNA polymerase. Sofosbuvir and Remdesivir as displayed in Figure 2, are such examples of pro-drugs. These are RNA polymerase inhibitors and are under clinical trials to treat COVID-19 (Holshue et al., 2020a; Wang et al., 2020a). The corona virus enzyme is able to accept modified nucleotide analogues due to its error-prone nature. Thus, nucleosides and nucleotides which inhibit polymerase are an important class of antiviral agents (Oberg, 2006; Eltahla et al., 2015). For future perspective, RNA-polymerases inhibitors are promising.

### 7.8. Failover

It shows anti-COVID-19 activity by blocking the enzyme the virus needs to survive in the host^11^11https://www.pharmaceutical-technology.com/news/china-approves-favilavir-covid-19/
.

### 7.9. Chloroquine and Remdesivir

These two drugs are antimalaria drugs and currently under assessment in more than 100 patients at over ten hospitals in Beijing and Guangdong provinces. Chloroquine prevents viral infections by increasing the endosome pH required for virus/cell fusion and interfering with the glycosylation COVID-19 cell receptors. A study by NIH officials confirms that the positive effect of Remdesivir drug against the MERS-CoV and COVID-19^12^12Antimalarial drug confirmed effective on COVID-19. [online] Website: http://www.xinhuanet.com/english/2020-02/17/c_138792545.htm [accessed 7 May 2020]
1313Preclinical data of Gilead’s remdesivir raises hope for China trials. [online] Website: https://www.pharmaceutical-technology.com/news/nih-gilead-remdesivir-monkey-data/ [accessed 7 May 2020]
, (Holshue et al., 2020b).

### 7.10. HIV medication

Two existing drugs, Ritonavir and Lopinavir, are used in combination for HIV but due to lack of a specific drug for COVID-19, Chinese health authorities recommended these drugs^14^14Results from Covid-19-focused trials in China may come within weeks. [online] Website: https://www.clinicaltrialsarena.com/news/china-covid-19-trials/ [accessed 7 May 2020].

### 7.11. Favipiravir

It is an approved drug for influenza, which inhibits the RNA-dependent RNA polymerase of RNA viruses, and a recent study reported its activity against COVID-19 (De Clercq, 2019; Wang et al., 2020b). 

### 7.12. Ribavirin

It is an approved drug for treating hepatitis C virus (HCV) and respiratory syncytial virus (RSV). Previously, Ribavirin was used in SARS and MERS patients (Zumla et al., 2016) and it may offer sufficient potency against COVID-19.

### 7.13. Disulfiram

It is an approved drug for alcohol addiction. It has been reported to inhibit the papain-like protease of MERS and SARS in in vitro but in vivo evidence is not available. At this critical situation, Disulfiram drug can be tested against COVID-19.

### 7.14. Hydroxychloroquine

It is used to cure patients with a disease like lupus, malaria, and rheumatoid arthritis for decades. Earlier in several studies, due to its antiviral activity and its potential to amend the immune system activity of Hydroxychloroquine alone or in combination with Azithromycin, it was believed that it might also be efficacious in COVID-19 treatment. However, the latest study evaluating data from 368 COVID-19 patients treated with Hydroxychloroquine alone or in combination with Azithromycin antibiotics show no benefits; instead, higher death rates were reported in the patients who were taking these medications. As per the data, 97 patients who took Hydroxychloroquine had a 27.8% death rate and the remaining 158 patients who did not take the drug had 11.4% of death rate (Magagnoli et al., 2020). Hence, these drugs still need to be studied before their widespread adoption.

Besides the drugs above, a list of anticoronavirus agents (in preclinical stage) is given in Table.

**Table:** List of antiviral compounds against human coronaviruses.

S.No	Antiviral agents	Drug targets/Mechanism	References
Virus-based treatment strategies			
1	Penciclovir	Inhibits RdRp	(Wang et al., 2020c)
2	Galidesivir	Inhibits viral RNA polymerase function	(Warren et al., 2014)
3	Thiopurine analogues	Inhibits PLpro	(Cheng et al., 2015)
4	Lopinavir	Inhibits 3CLpro	(Arabi et al., 2018; Kim et al., 2015; Sheahan et al., 2020)
5	Ritonavir	Inhibits 3CLpro	(Arabi et al., 2018; Kim et al., 2015; Sheahan et al., 2020)
6	Darunavir and cobicistat	Inhibits 3CLpro	( Lu, 2020)5
7	ASC09F (HIV protease inhibitor)	Inhibits 3CLpro	(Xiaowei Xu, Jian Liu, 2020)16
8	Nafamostat	Inhibits spike-mediated membrane fusion	(Ito et al., 2016; Wang et al., 2020b)
9	Griffithsin	Griffithsin binds to the SARS-CoV spike glycoprotein, thus inhibiting viral entry	(Barton et al., 2014; O’Keefe et al., 2010)
10	Arbidol (Umifenovir)	Not known	(Burke, 17 FEB)17
11	Oseltamivir	Oseltamivir is an influenza neuraminidase inhibitor.	(Huang et al., 2020)
Host-based treatment strategies			
12	Recombinant Interferons (a & b)	Exogenous interferons	(Falzarano et al., 2013; Kim et al., 2015; O’Keefe et al., 2010)
13	Nitazoxanide	Induces the host innate immune response to produce interferons (a & b) by the host’s fibroblasts and protein kinase R (PKR) activation	(Rossignol, 2014)
14	Cyclosporine A	Cyclophilin inhibitor	(Pfefferle et al., 2011)
15	Alisporivir	Cyclophilin inhibitor	(de Wilde et al., 2017b)
16	Selumetinib	Inhibits the ERK/MAPK and PI3K/AKT/mTOR signalling pathways	(Kindrachuk et al., 2015)
17	Trametinib	Inhibits the ERK/MAPK and PI3K/AKT/mTOR signalling pathways	(Kindrachuk et al., 2015)
18	Rapamycin	Inhibits the ERK/MAPK and PI3K/AKT/mTOR pathways significantly inhibited MERS-CoV replication	(Kindrachuk et al., 2015)
19	K11777, Camostat	Blocks endosomal protease-mediated cleavage and the endosomal entry pathway	(Zhou et al., 2015)
20	Mycophenolic acid	Inhibits IMPDH and guanine monophosphate synthesis	(Hart et al., 2014)
21	Silvestrol	Inhibits the DEAD-box RNA helicase eIF4A to affect virus translation	(Müller et al., 2018)

^15^Lu H (2020). Efficacy and Safety of Darunavir and Cobicistat for Treatment of COVID-19 (DC-COVID-19). [online] Website: https://clinicaltrials.gov/ct2/show/record/NCT04252274 [accessed 7 May 2020] ^16^Xu X, Liu J (2020). Evaluating and Comparing the Safety and Efficiency of ASC09/Ritonavir and Lopinavir/Ritonavir for Novel Coronavirus Infection. [online] Website: https://clinicaltrials.gov/ct2/show/NCT04261907 [accessed 7 May 2020] ^17^Burke CW (2020). Mobilizing drug development efforts against the novel coronavirus. [online]. Website: https://www.biospace.com/article/mobilizing-drug-development-efforts-against-the-novel-coronavirus/ [accessed 7 May 2020].

## 8. Prevention and treatment

It is well said that “prevention is better than cure.” We do know that COVID-19 is an infectious disease outbreak. Since it is a new virus, there is currently no vaccine or specific treatment, and the understanding of the virus and the disease are still evolving. Currently, in the absence of any proven therapy for COVID-19, the mainspring care for the infected patients is still supportive care from symptomatic outpatient management to full intensive care support. The most efficacious abiding strategy for prevention of COVID-19 outbreak in the future would be the development of a vaccine to avert the infection and to provide protective immunity among people. Many research groups have designed potential vaccines and are in clinical phase; however, there is much more work to do. Thus, for now, we need to apply basic cautions like hand hygiene, coughing etiquette, putting a mask on face, avoiding close contact with people showing symptoms of respiratory diseases, and inform a doctor when feeling sick. Many countries imposed stay-at-home orders or lockdowns as a preventive measure of the further transmission of the infection, as quarantine and social distancing is a very notable factor to cease the transmission. Hence, it is recommended to avoid large crowded public places and to maintain at least 6 feet of distance between yourself and others, especially if you are around ill people. People with older age and those with comorbidities need extra care, and they should also avoid unnecessary travel. Frequent hand washing for at least 20 s is the best way to protect yourself and your loved ones.

## 9. The of COVID-19 on the global economy

COVID-19 had a major impact on the global economy throughout the first quarter of 2020. More than 210 countries are affected by this pandemic leading to almost half of the world population on lockdown to prevent the spread of deadly COVID-19 virus. The Economist forecasts the slowing of the global economy by 6% in 2020, 0.7% of growth, and an average of 1.2% contraction. Countries which are experiencing the economic shock of COVID-19 pandemic include US (–5.9%), Japan (–5.2%), UK (–6.5%), Germany (–7.0%), France (–7.2%), Italy (–9.1%), Spain (–8.0%). Also, the Central Asian Countries and Middle East countries will be shrunk by 2.8% with Saudi Arabia’s growth forecast at –2.3%. As the world’s largest manufacturing economy, China evolves its manufacturing capabilities to supply products from low-cost goods to more advanced ones globally has now been decelerated due to this vicious viral outbreak. As the factories in most of the countries are still shut due to the outbreak and also due to unavailability of transport, travel, and labor supply because of restriction on movement and social distancing which eventually results in disrupted supply and lost revenue in business. And now, there has been the complete shutdown of all sectors, including nonessential retail trade, hotels, restaurants, and tourism. The disruption of the supply chain and unavailability of medical supplies and drugs around the world could be seen because of the Chinese dominance in the pharmaceutical market as it is the dominant and often the only global supplier for the active ingredients of some vital medication, medical devices, and equipment. 

The tourism industry is currently one of the most affected sectors by COVID-19, which holds 10% of global GDP. The pandemic could affect more than 50 million jobs globally in this industry. As a result of flight cancellations, the global airline revenues are expected to dive by 4–5 billion $ in the first quarter of 2020. As per the forecast by the UN’s International Civil Aviation Organization (ICAO) and World Travel and Tourism Council (WTTC), International travel could be adversely struck by up to 25% in the year 2020. Also, due to lockdown and travel restrictions, the demand for oil has reduced worldwide. The unprecedented drop in oil price below zero $ for the first time in history has been experienced due to the sapped demand for oil because of the COVID-19 pandemic. The catastrophic imbalance of oversupplied oil in the market causes the West Texas Intermediate (WTI), a global benchmark for oil prices, costs less than zero dollars on April 19, 2020.

## 10. Discussion

Coronavirus disease, which started in the last month of 2019, has spread its feet almost all over the world. It hit every sector of the world, from a survival level to large scale business, created fear of death and uncertainty. It has forced the world to stay at home. Since the beginning of its outbreak with the infection until 06 May 2020, more than infected cases with deaths around the globe have been reported. The initial infected cases of COVID-19 were directly or indirectly linked to Huanan seafood and wild animal wholesale market, where it was possibly transmitted through bats. After all, now it has expanded its modes of transmission like, through animals, human to human, and fecal transmission. After inhaling into the body it takes around 2 to 14 days to incubate, and the journey begins with normal symptoms like cough, fever, etc. to severe illness and in the end death due to weakness of immunity. Some other factors are also responsible for increasing death rate like lifestyle, age (main factor), and weak immune system due to having another disease. Among more than 210 countries the report says developed countries are most affected by this pandemic. It has been seen that COVID-19 may be far more infectious in comparison to other flu infections, because R0 value is lower than other viruses, like chickenpox, mums, and SARS. 

The whole world is currently engaged in research on COVID-19 to control it. A genetic study has revealed its sequence similarity with previous with SARS and MERS. The whole-genome sequence of SARS-CoV-2 has been identified which will provide more options for the development of diagnosis and treatment. It is very unfortunate for us that there is no medicine or vaccine available yet although several research institutes and drug manufacturer companies have announced to develop vaccines. The drug repurposing has emerged as a basic clinical tool to find new molecular entities having an impact on treating associated disease. The existing drugs help to reduce the cost of development and time and they have a higher rate of success. Research on a number of existing drugs having antiviral activity is underway across the world.

After starting in 2019, this pandemic continued to spread around the world. And this put the world in a big financial crisis. The Economist reported a 6% slowdown of the world’s economy in this year, 0.7% of growth and the average 1.2% contraction. It is still difficult to estimate how much more this will affect the economy; this will definitely depend on how long it will take to discover a vaccine or drug.

## 11. Conclusion

The previous epidemic in the 19th–20th century and now this COVID-19 made people and governments all over the world realize the importance of public health and now how this could be a key factor in improving the global life expectancy in the near future. The economic disruption and discontinuation of the manufacturing supply chain of goods across the world are indeed a matter of great concern too because the varying per day new cases of COVID-19 across the globe painfully exposes the current and persisting health inequalities in our society and emphasizes the crucial necessity of collective action of various countries, institutions, academics, and pharmaceutical companies against this outbreak across the world. However, public health bodies should keep an eye on the situation closely, as the more we know about this deadly virus and its associated outbreak, the better we can respond.

## Acknowledgments

RI and AA are grateful to the Centre for Interdisciplinary Research in Basic Sciences (CIRBSc), Jamia Millia Islamia-110025 for providing the research infrastructure. AA acknowledges the Department of Health Research (DHR) New Delhi, India for the award of “Young Scientist” fellowship (R.12014/06/2019-HR). NI is thankful to Indian Council of Medical Research (ICMR), New Delhi, India. MFS is grateful to Prof. Muratov Zhanybek Kudaibakovich and Dr. Syed Ali Abbas from Osh State University, Kyrgyzstan for their guidance and support.

## Contribution of authors

AA and MFS contributed equally to this work. RI and AA conceived the study design, instructed on data analysis, and drafted the manuscript. MFS, RA, MM, and NI curated data and drew the figures. All the authors read, edited, and approved the manuscript.

**Table:** Abbreviations

WHO	World Health Organization	CDC	Center For Disease Control and Prevention
COVID-19	Coronavirus Disease-2019	HCoVs	Human Coronaviruses
HBV	Hepatitis B Virus	MERS	Middle East Respiratory Syndrome
HCoV	Human Coronavirus	MERS-CoV	Middle East Respiratory Syndrome Coronavirus
HCV	Hepatitis C Virus	ARDS	Acute Respiratory Distress Syndrome
IAV	Influenza A Virus	PLpro	Papain-Like Protease
SPHCC	Shanghai Public Health Clinic Center	ICTV	International Committee on Taxonomy of Viruses
NCIP	Novel Corona Virus-Infected Pneumonia	MOF	Multiple Organ Failure
TCM	Traditional Chinese Medicine	LAIV	Live-Attenuated Investigational Vaccine
WTTC	World Travel and Tourism Council	ICAO	International Civil Aviation Organization
RdRp	RNA-Dependent RNA Polymerase	RSV	Respiratory Syncytial Virus
SARS-CoV	Severe Acute Respiratory Syndrome Coronavirus	IEA	International Energy Agency
